# Supplementary material for: How strongly does diet variation explain variation in isotope values of animal consumers?
Source: PLoS One. 2024 Jun 27;19(6):e0301900. doi: 10.1371/journal.pone.0301900 (PMC11210776; doi:10.1371/journal.pone.0301900)
Supplement: S1 File — Derivations of the general and generic predictions. (PDF) [file pone.0301900.s001.pdf]

Derivations of the general and generic predictions

July 31, 2023

Jean-François Arnoldi[1*], Jenny Rose Bortoluzzi[2], Hugh Rowland, Chris Harrod[3,4,5], Andrew Parnell[6], Nicholas Payne[2], Ian Donohue[2], Andrew Jackson[2],

1 Theoretical and Experimental Ecology Station, CNRS Moulis, Moulis, France
2 Department of Zoology, School of Natural Sciences, Trinity College Dublin, Dublin 2, Ireland.
3 Instituto de Ciencias Naturales Alexander von Humboldt, Universidad de Antofagasta, Avenida Angamos 601, Antofagasta, Chile
4 Instituto Antofagasta, Universidad de Antofagasta, Avenida Angamos 601, Antofagasta, Chile
5 Millennium Nucleus of Austral Invasive Salmonids (INVASAL), Concepciòn, Chile
6 Insight Centre for Data Analytics, Hamilton Institute, Maynooth University, Kildare, Ireland

* Jean-Francois.Arnoldi@cnrs.fr

Proof of the general claim: For simplicity we drop the index μ and the additive term ψ. Given that the vector-valued random variables $\boldsymbol{f}$ and $\boldsymbol{x}$ *are assumed independent*:

$$\bar{y} = \langle \bar{\boldsymbol{f}}, \bar{\boldsymbol{x}} \rangle$$

and

$$\mathrm{var}(y) = \overline{\langle \boldsymbol{x}, \boldsymbol{f} \rangle \langle \boldsymbol{f}, \boldsymbol{x} \rangle} - \langle \bar{\boldsymbol{x}}, \bar{\boldsymbol{f}} \rangle \langle \bar{\boldsymbol{f}}, \bar{\boldsymbol{x}} \rangle$$

Using Dirac's "bra-ket" notations we can rewrite this expression as a trace of matrices

$$= \mathrm{Tr}\left(\overline{|\boldsymbol{x}\rangle \langle \boldsymbol{x}|} \times \overline{|\boldsymbol{f}\rangle \langle \boldsymbol{f}|} - |\bar{\boldsymbol{x}}\rangle \langle \bar{\boldsymbol{x}}| \, |\bar{\boldsymbol{f}}\rangle \langle \bar{\boldsymbol{f}}| \right)$$

and note that

$$\overline{|\boldsymbol{x}\rangle \langle \boldsymbol{x}|} = C_x + |\bar{\boldsymbol{x}}\rangle \langle \bar{\boldsymbol{x}}| \, ; \; \overline{|\boldsymbol{f}\rangle \langle \boldsymbol{f}|} = C_f + |\bar{\boldsymbol{f}}\rangle \langle \bar{\boldsymbol{f}}|$$

where $C_{x,f}$ are the covariance matrices of $\boldsymbol{f}$ and $\boldsymbol{x}$. So, it holds that

$$\overline{|\boldsymbol{x}\rangle \langle \boldsymbol{x}|} \times \overline{|\boldsymbol{f}\rangle \langle \boldsymbol{f}|} - |\bar{\boldsymbol{x}}\rangle \langle \bar{\boldsymbol{x}}| \, |\bar{\boldsymbol{f}}\rangle \langle \bar{\boldsymbol{f}}|$$

$$= \overline{|\boldsymbol{x}\rangle \langle \boldsymbol{x}|} \left(C_f + |\bar{\boldsymbol{f}}\rangle \langle \bar{\boldsymbol{f}}| \right) - |\bar{\boldsymbol{x}}\rangle \langle \bar{\boldsymbol{x}}| \, |\bar{\boldsymbol{f}}\rangle \langle \bar{\boldsymbol{f}}|$$
$$= \overline{|\boldsymbol{x}\rangle \langle \boldsymbol{x}|} C_f + C_x \, |\bar{\boldsymbol{f}}\rangle \langle \bar{\boldsymbol{f}}|$$
$$= C_x C_f + |\bar{\boldsymbol{x}}\rangle \langle \bar{\boldsymbol{x}}| \, C_f + C_x \, |\bar{\boldsymbol{f}}\rangle \langle \bar{\boldsymbol{f}}|$$

Using the additivity of the trace we arrive to

$$\mathrm{var}(y) = \mathrm{Tr}\left(C_x C_f \right) + \langle \bar{\boldsymbol{x}}| \, C_f \, |\bar{\boldsymbol{x}}\rangle + \langle \bar{\boldsymbol{f}}| \, C_x \, |\bar{\boldsymbol{f}}\rangle$$

Because of the sum-to-one property of diet vectors, the vector $\boldsymbol{f} - \bar{\boldsymbol{f}}$ sums to zero, meaning that it is orthogonal to the vector $\mathbf{1}$ (which is the normal vector to the surface of the simplex). Because of this property the covariance matrix C_f is of rank $S-1$ with a zero eigenvalue in the direction of vector $\mathbf{1}$. Importantly, this implies that

$$\langle \bar{\boldsymbol{x}} | C_f | \bar{\boldsymbol{x}} \rangle = \langle P\bar{\boldsymbol{x}} | C_f | P\bar{\boldsymbol{x}} \rangle$$

where P is the projector defined for any vector u as $Pu = u - \frac{\langle u, \mathbf{1} \rangle}{S}\mathbf{1}$. We now use the spectral decomposition of the matrices $C_x = \sum \sigma_i^2 |u_i\rangle \langle u_i|$ and $C_f = \sum \lambda_j^2 |v_j\rangle \langle v_j|$ where the eigenvectors $\{u_i\}$ and $\{v_j\}$ both form orthonormal basis of $\mathbb{R}^S$ to show that

$$\mathrm{Tr} C_x C_f = \sum_{i,j} \sigma_i^2 |\langle u_i, v_j \rangle|^2 \lambda_j^2 \leq \sum_{i,j} \sigma_i^2 \lambda_j^2 = \sum_i \sigma_i^2 \sum_j \lambda_j^2 = \mathrm{Tr} C_x \mathrm{Tr} C_f$$

and

$$\langle P\bar{\boldsymbol{x}} | C_f | P\bar{\boldsymbol{x}} \rangle = \sum_j \lambda_j^2 |\langle P\bar{\boldsymbol{x}}, v_j \rangle|^2 \leq ||P\bar{\boldsymbol{x}}||^2 \sum_j \lambda_j^2 = ||P\bar{\boldsymbol{x}}||^2 \mathrm{Tr} C_f$$

and similarly

$$\langle \bar{\boldsymbol{f}} | C_x | \bar{\boldsymbol{f}} \rangle \leq ||\bar{\boldsymbol{f}}||^2 \mathrm{Tr} C_x$$

Taken together these inequalities lead us to

$$\mathrm{var}(y) \leq \mathrm{Tr} C_x \mathrm{Tr} C_f + ||P\bar{\boldsymbol{x}}||^2 \mathrm{Tr} C_f + ||\bar{\boldsymbol{f}}||^2 \mathrm{Tr} C_x$$

We note that

$$\mathrm{Tr}\,(C_f) = \overline{||\boldsymbol{f}||^2} - ||\bar{\boldsymbol{f}}||^2$$

using this relationship we get to

$$\mathrm{var}(y) \leq \overline{||\boldsymbol{f}||^2} \left(\mathrm{Tr}\,(C_x) + ||P\bar{\boldsymbol{x}}||^2 \right) - ||\bar{\boldsymbol{f}}||^2 ||P\bar{\boldsymbol{x}}||^2$$

If we define

$$\phi = \frac{||P\bar{\boldsymbol{x}}||^2}{||\mathrm{std}(\boldsymbol{x})||^2 + ||P\bar{\boldsymbol{x}}||^2}$$

then

$$\mathrm{var}(y) \leq \left(||\mathrm{std}(\boldsymbol{x})||^2 + ||P\bar{\boldsymbol{x}}||^2 \right) \left(\overline{||\boldsymbol{f}||^2} - \phi ||\bar{\boldsymbol{f}}||^2 \right)$$

but given that

$$\overline{||\boldsymbol{f}||^2} - \phi ||\bar{\boldsymbol{f}}||^2 \leq 1 - \frac{\phi}{S}$$

this suggests a normalization by $1 - \frac{\phi}{S}$, which gives

$$\frac{\mathrm{var}(y)}{\mathrm{baseline}} \leq \frac{S}{S - \phi} \left(\overline{||\boldsymbol{f}||^2} - \phi ||\bar{\boldsymbol{f}}||^2 \right) \leq 1$$

where the absolute baseline is identified as

$$\mathrm{baseline} = ||\mathrm{std}(\boldsymbol{x})||^2 + \frac{S-1}{S} ||P\bar{\boldsymbol{x}}||^2$$

Proof of the generic claim: Above we assumed independent random variables but ask nothing in particular about the relationships between their moments, i.e. their covariance matrices $C_{x,f}$ and the means $\bar{\boldsymbol{x}}$ and $\bar{\boldsymbol{f}}$. We now deduce an expectation based over a random universe of such objects. Our results are based on the following feature of independent random vectors in $\mathbb{R}^d$ whose directions are equiprobable

$$\mathbb{E}\left|\langle \boldsymbol{u}, \boldsymbol{v}\rangle\right|^2 = \frac{\mathbb{E}||\boldsymbol{u}||^2\mathbb{E}||\boldsymbol{v}||^2}{d}$$

Using this we will show that

- $\mathrm{Tr}\left(C_x C_f\right) \approx \frac{1}{S}\mathrm{Tr}\left(C_x\right)\mathrm{Tr}\left(C_f\right)$
- $\mathrm{Tr}\left(\left|\bar{\boldsymbol{x}}\right\rangle\left\langle\bar{\boldsymbol{x}}\right| C_f\right) = \left\langle P\bar{\boldsymbol{x}}\right| C_f \left|P\bar{\boldsymbol{x}}\right\rangle \approx \frac{||P\bar{\boldsymbol{x}}||^2}{S-1}\mathrm{Tr}\left(C_f\right)$
- $\mathrm{Tr}\left(C_x\left|\bar{\boldsymbol{f}}\right\rangle\left\langle\bar{\boldsymbol{f}}\right|\right) = \left\langle\bar{\boldsymbol{f}}\right| C_x \left|\bar{\boldsymbol{f}}\right\rangle \approx \frac{||\bar{\boldsymbol{f}}||^2}{S}\mathrm{Tr}\left(C_x\right)$

For the first point we use the spectral decomposition of the covariance matrices

$$\mathrm{Tr}\left(C_x C_f\right) = \sum_{i,j}\sigma_i^2\left|\langle u_i, v_j\rangle\right|^2\lambda_j^2$$

and then replace $\left|\langle u_i, v_j\rangle\right|^2$ by its expected value $1/d$ with $d = S$ if the directions of these vectors where independent and uniformly drawn. The third point follows in the same manner while for the second point we use the additional fact that the vectors $P\bar{\boldsymbol{x}}$ and the eigenvectors of C_f associated to non-zero eigenvalues lie in a subspace of dimension $d = S - 1$. We then arrive at

$$\mathrm{var}(y) \approx \overline{\|\boldsymbol{f}\|^2}\left(\frac{||\mathrm{std}(\boldsymbol{x})||^2}{S} + \frac{||P\bar{\boldsymbol{x}}||^2}{S-1}\right) - \left\|\bar{\boldsymbol{f}}\right\|^2\frac{||P\bar{\boldsymbol{x}}||^2}{S-1}$$

and if we define

$$\phi^* = \frac{||P\bar{\boldsymbol{x}}||^2}{||P\bar{\boldsymbol{x}}||^2 + \frac{S-1}{S}||\mathrm{std}(\boldsymbol{x})||^2}$$

then

$$\frac{\mathrm{var}(y)}{\mathrm{baseline}^*} \approx \frac{S}{S-\phi}\left(\overline{\|\boldsymbol{f}\|^2} - \phi\left\|\bar{\boldsymbol{f}}\right\|^2\right) \leq 1$$

where

$$\mathrm{baseline}^* = \frac{||\mathrm{std}(\boldsymbol{x})||^2 + ||P\bar{\boldsymbol{x}}||^2}{S}$$
