# Supplementary material for: How strongly does diet variation explain variation in isotope values of animal consumers?
Source: PLoS One. 2024 Jun 27;19(6):e0301900. doi: 10.1371/journal.pone.0301900 (PMC11210776; doi:10.1371/journal.pone.0301900)
Supplement: Attachment — Submitted filename: Review of PONE D 23 24341.pdf [file pone.0301900.s002.pdf]

Review of PONE D 23 24341

By Arnoldi et al.

The paper aims at determining if the variation in consumer stable isotope values reflects the ones of the diet using in silico simulation approaches. Authors showed (under very strong assumptions) that the answer is yes once considered the intra and inter variance of the isotopic baseline. The paper is well written, and i like very much the idea of testing the caveats of isotopic ecology using maths as already done by Flynn et al., (2018 Mar Biol), Jabot et al., (2017 Funct Ecol) or Ballutaud et al. (2022 Plos one) for instance. I cannot judge fully on the mathematics developped here (I am not mathematician and there is heavy stuff in this paper) but i can say with high certitude that a common isotope ecologist will be completely overwhelmed by the mathematical developments done in this paper. At least, i advice the authors to apply their findings to a case study as done by Yeakel et al. (2016).

Some more specific comments below.

The submission suffers from the absence of line numbering that would have eased the review process

Intro

- First paragraph of the intro is about ecological niche while the reader would be much more interested in the trophic niche. Please clarify or explain.
- The statement « often difficult to observe organism's niche » is not supported by any reference and is not clear at all. There is a bunch of literature on ecological niche. Please clarify and be specific regarding the trophic niche (stomach content may help and is observable in the field?)
- Second paragraph « Turnover rates which vary depending on the animal ». Authors means species or individual or both ? Also again possible confusion between ecological and trophic niche. Trophic niche is a sub set of the ecological niche ?

Model

- « Earlier than 1-5/r » is coming out from the blue. Please demonstrate. Ballutaud et al., (2022 plos one) estimated that the « 5/r » would be equal to 2*ln(2)/ln(r)
- « As long as diet temporal fluctuations are not too strongly correlated…. ». Could you provide a threshold in the correlation? Is it realistic in nature? This is an extremely important assumption which is not acknowledged anymore in the rest of the manuscript.
- Going from eq 3 to eq 4 is extremely harsh !
- I suspect that an additional assumption must be made : All individual must have the same turnover rate. Otherwise another source of variation must be accounted for. If correct please add. If not please argue.

Result and discussion

- Page 9 « simplifying assumptions » please remind them + the one on turnover rate
- Phillips 2014 and Gorokhova 2018 are not in the reference list

Conclusion

- « We nonetheless expect to see a quantitative relationship » sounds like a weak conclusion. I prefer the « semi-quantative comparisons » terms. For example, Jabot et al. (2017 functional

ecology) wrote « researchers should not hope to grasp subtle patterns of food web structure based solely on widely used isotopic indices. The remaining question is more about quantifying the thresholds (the limits as written by the authors) when variance in isotope values of the consumer does not reflect variance in the diet. No conclusions were drawn on these limits whereas this is a key word of the MS title. As a title i would prefer the sentence at the end of intro « how strong is the signal of diet …. » or better « How strengthen the signal of diet …. »
